# Multiple Contact Dates and SARS Incubation Periods

**DOI:** 10.3201/eid1002.030426

**Published:** 2004-02

**Authors:** Martin I. Meltzer

**Affiliations:** * Centers for Disease Control and Prevention, Atlanta, Georgia, USA

**Keywords:** severe acute respiratory syndrome, incubation period, multiple contact dates, estimation, spreadsheet

## Abstract

Many severe acute respiratory syndrome (SARS) patients have multiple possible incubation periods due to multiple contact dates. Multiple contact dates cannot be used in standard statistical analytic techniques, however. I present a simple spreadsheet-based method that uses multiple contact dates to calculate the possible incubation periods of SARS.

The appearance and rapid spread of severe acute respiratory syndrome (SARS), caused by a previously unknown coronavirus (SARS-CoV) (*1*–*3*), has already had a notable economic and social impact (*4*,*5*). SARS has no definitive cure, although hospitalized patients have been empirically treated with combinations of antibiotics, steroids, antiviral drugs (typically ribavirin and oseltamivir), and mechanical ventilation (*6*,*7*). No known drug can be used prophylactically, nor is does a vaccine exist. Thus, to stop the spread of the disease, public health officials have to rely almost completely on placing those who may have been exposed to SARS-CoV under quarantine and isolating those with suspected, probable, and confirmed SARS cases.

To make quarantine and isolation as effective as possible, knowing the range of the possible incubation period of SARS is essential. Mathematical modelers also need to know the characteristics of the incubation period to provide estimates of possible spread and model the potential impact of interventions. Many SARS patients often report more than one possible date of contact with another known SARS patient (*6*,*7*), however, which results in multiple dates of possible transmission and infection (Table). These multiple dates prevent easily identifying a discrete period of incubation for each patient, and thus the data from such patients cannot be used in standard statistical analytic techniques, such as regression analyses (unless the analyst chooses a single incubation period from the possible choices) (*8*).

**Table:** Patients with severe acute respiratory syndrome (SARS) and possible incubation periods

Patient source and no.^a^	Possible incubation period of SARS in days																	
	1	2	3	4	5	6	7	8	9	10	11	12	13	14	15	16	17	18
Canada 1		2	3	4	5	6	7	8	9	10	11	12						
Canada 2	1	2	3	4														
Canada 3	1			4														
Canada 4	1	2	3	4	5	6	7	8	9	10	11							
Canada 5	1	2	3	4	5	6	7	8	9	10	11	12	13	14				
Canada 7			3							10								
Canada 8^b^			3															
Canada 10	1	2	3	4	5	6												
Hong Kong 2		2																
Hong Kong 3		2																
Hong Kong 4						6												
Hong Kong 5		2																
Hong Kong 6	1	2	3	4	5	6												
Hong Kong 7					5	6	7	8	9	10	11							
Hong Kong 8					5	6	7	8	9	10	11							
Hong Kong 9	1	2	3	4	5													
Hong Kong 10		2	3	4	5	6	7											
USA 1						6							13	14	15	16	17	18
USA 2							7	8	9	10	11	12						

^a^Patient source: Canada refers to patients reported in reference 6, Hong Kong to patients reported in reference 7, and USA to patients whose incubation periods were extracted from an unpublished database held at CDC. I used the same patient numbers as used in the published reports. ^b^Patient 9 from the Canadian database (*6*) was excluded because the possible incubation period was reported as < 29 days. However, even with n = 20, adding patient Canada 9 would mean that possible incubation periods between 19 and 29 days would each have very low frequencies (i.e., <0.01).

I present a simple method that allows a simulation of the frequency distribution, including confidence intervals, of the possible incubation periods (in days) for SARS. The method allows use of data from patients with multiple potential incubation periods. One goal of the method was to keep it simple by using common computer spreadsheet software, allowing for easy replication, extension of the database and results, and rapid dissemination of the method. The method can also be used to calculate when infectious persons are most likely to have transmitted SARS to susceptible persons, even when multiple days of possible transmission exist.

## Methods

I used published data reporting possible incubation periods for 17 patients (*6*,*7*) plus data from two case-patients in an unpublished database maintained at the Centers for Disease Control and Prevention (CDC). The data illustrate a common problem: many patients have multiple possible incubation periods. I built a simulation model in a standard computer spreadsheet (Excel 2000, Microsoft Corp, Redmond, WA) (See Appendix). I first listed each possible incubation period for every patient for whom incubation period data were available (Table). Then, for every patient, I assigned a random number generator (function RAND in Excel software) to each possible incubation period. This method is the equivalent of using a uniform distribution to select an incubation period from all possible choices. Using a spreadsheet-based simulation software package (@Risk, Palisade Corp., Newfield, NY), I programmed the spreadsheet to run iterations of the model.

During a single iteration, for each patient, the programmed model selects the incubation period with the highest random number for that iteration. After a single iteration, the program calculates the frequency distribution for the incubation periods. Then, the program assigns another set of random numbers to each possible incubation period and selects and calculates the frequency distribution. After numerous iterations, the program combines all the frequency distributions from all iterations to provide a general frequency distribution. From this final frequency distribution, descriptive statistics can be obtained, such as the mean, median, 5th and 95th percentile values. I ran approximately 10,000 iterations, at which point each additional iteration caused the mean and the standard distribution for each possible day of incubation to change by <1%.

## Results

**Figure 1:**
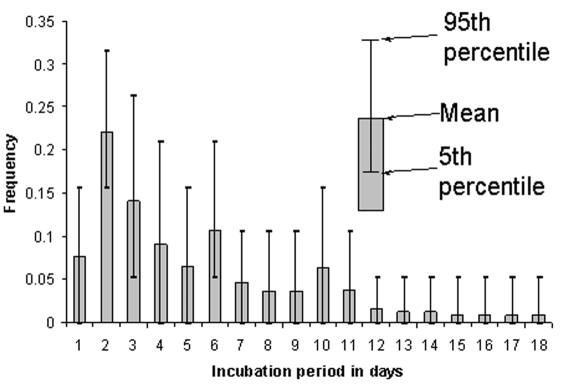
Simulation of frequency distribution of incubation period of severe acute respiratory syndrome. Data used for this simulation were obtained from Canada (*6*), Hong Kong (*7*), and the United States, for a total sample size of 19. Many of the patients included in the database had multiple possible incubation periods (see Table), resulting in the confidence intervals displayed for each day.

**Figure 2:**
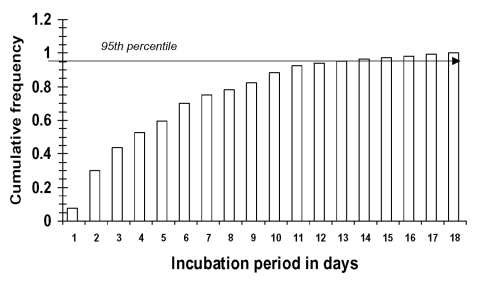
Cumulative frequency incubation period of severe acute respiratory syndrome. Data are the mean frequencies of each individual incubation period, as shown in Figure 1. Data used for this simulation were obtained from Canada (*6*), Hong Kong (*7*), and the United States, for a sample size 19. Many of the patients included in the database had multiple possible incubation periods (see Table).

The three largest mean frequencies of incubation periods among the patients examined were 2, 3, and 6 days (Figure 1). Incubation periods of 1, 4, 5, and 10 days were the second highest mean frequencies (Figure 1). However, the confidence intervals (5th and 95th percentiles) for most of the potential incubation periods clearly overlapped (Figure 1). This finding indicates that with the given data set, an incubation period of 10 days is almost as likely to occur as an incubation period of 6 days. Using the mean frequency of each incubation period, I constructed a cumulative frequency graph (Figure 2). The 95th percentile is 12 days, with a median (50th percentile) of approximately 4 days.

## Discussion

The incubation period for SARS is likely to be varied, with the frequency distribution being nonnormal (Figure 1). Thus, using mean incubation periods for activities such as mathematical modeling will probably result in a misrepresentation of SARS transmission. The type of analysis presented here can help public health officials determine minimum quarantine periods for persons exposed to SARS, who are not yet symptomatic. For example, public health authorities should be aware that in a small percentage of case-patients, the incubation period might be >10 days (Figure 2).

Given that data from only 19 patients were available for this analysis, some caution should be exercised when evaluating the results. Adding or subtracting relatively small numbers of patients can cause estimates such as the 95th percentile of the cumulative frequency to change. More data concerning the possible incubation period of SARS patients are needed. The advantage of the method used here is that such data need not be specific. The method readily “accepts” data in which patients have multiple possible incubation periods. More data will likely reduce the confidence intervals for the frequencies of each incubation day (Figure 1), giving a clearer picture of the actual frequency distribution of all incubation periods.

The method can also be readily adapted to examine other aspects of SARS epidemiology when unambiguous data are scarce. For example, with the appropriate data, this method can be used to examine the frequency distribution of when an infectious person infects other people. (An Excel workbook [Excel 2000, Microsoft, Corp, Redmond, WA] containing the model used to calculate the results shown in Figures 1 and 2, and using the data shown in the Table, is available in the Appendix). Also, distributions of incubation periods can be used to examine whether an association exists between incubation period and likelihood of hospitalization or death.

## Supplementary Material

Appendix Spreadsheet model to calculate incubation period of SARS
